# Light-Limited Growth Rate Modulates Nitrate Inhibition of Dinitrogen Fixation in the Marine Unicellular Cyanobacterium *Crocosphaera watsonii*

**DOI:** 10.1371/journal.pone.0114465

**Published:** 2014-12-11

**Authors:** Nathan S. Garcia, David A. Hutchins

**Affiliations:** Marine and Environmental Biology, Department of Biological Sciences, University of Southern California, Los Angeles, CA, United States of America; CEA-Saclay, France

## Abstract

Biological N_2_ fixation is the dominant supply of new nitrogen (N) to the oceans, but is often inhibited in the presence of fixed N sources such as nitrate (NO_3_^−^). Anthropogenic fixed N inputs to the ocean are increasing, but their effect on marine N_2_ fixation is uncertain. Thus, global estimates of new oceanic N depend on a fundamental understanding of factors that modulate N source preferences by N_2_-fixing cyanobacteria. We examined the unicellular diazotroph *Crocosphaera watsonii* (strain WH0003) to determine how the light-limited growth rate influences the inhibitory effects of fixed N on N_2_ fixation. When growth (*µ*) was limited by low light (*µ* = 0.23 d^−1^), short-term experiments indicated that 0.4 µM NH_4_^+^ reduced N_2_-fixation by ∼90% relative to controls without added NH_4_^+^. In fast-growing, high-light-acclimated cultures (*µ* = 0.68 d^−1^), 2.0 µM NH_4_^+^ was needed to achieve the same effect. In long-term exposures to NO_3_^−^, inhibition of N_2_ fixation also varied with growth rate. In high-light-acclimated, fast-growing cultures, NO_3_^−^ did not inhibit N_2_-fixation rates in comparison with cultures growing on N_2_ alone. Instead NO_3_^−^ supported even faster growth, indicating that the cellular assimilation rate of N_2_ alone (i.e. dinitrogen reduction) could not support the light-specific maximum growth rate of *Crocosphaera*. When growth was severely light-limited, NO_3_^−^ did not support faster growth rates but instead inhibited N_2_-fixation rates by 55% relative to controls. These data rest on the basic tenet that light energy is the driver of photoautotrophic growth while various nutrient substrates serve as supports. Our findings provide a novel conceptual framework to examine interactions between N source preferences and predict degrees of inhibition of N_2_ fixation by fixed N sources based on the growth rate as controlled by light.

## Introduction

Understanding the global N cycle is critical to ocean biogeochemical models, as nitrogen is arguably the single most limiting nutrient for oceanic primary production. A major current challenge is to determine how N biogeochemistry will change as we transition from the Holocene to the Anthropocene [1]. Nitrogen fixation is one of the key pathways predicted to change as the surface ocean becomes warmer and more acidified [2], [3], [4], [5], [6], [7] and as progressive anthropogenic eutrophication increases fixed N loading in many marine ecosystems [8], [9].

Modeled estimates of N input from marine biological N_2_ fixation are dependent on concentrations of other chemical species of fixed N such as nitrate (NO_3_^−^) [10], [11]. This is largely because fixed N has been shown in past studies to have relatively strong “inhibitory” effects on N_2_-fixation by the ubiquitous oceanic diazotroph *Trichodesmium* [12], [13], [14], [15], most likely due to differences in the energetic costs involved in assimilating different N species such as NO_3_^−^ and N_2_ [16]. Several recent laboratory studies, however, have suggested that N_2_ fixation by unicellular diazotrophs such as *Crocosphaera watsonii* may not be as strongly inhibited by NO_3_^−^ as has been previously suggested for *Trichodesmium* [14], [15], [17].

While this major physiological difference may relate to differences in N_2_-fixation strategies (*Trichodesmium* fixes N_2_ during the day; *Crocosphaera* fixes N_2_ during the night, similar to unicellular organismal physiology described by Berman-Frank et al. [18]), these recent findings imply that the ratios of N-assimilation kinetic parameters for different N sources (e.g. V_max,N2_:V_max,NO3−_) may be very different between *Trichodesmium* and *Crocosphaera*. In addition to these laboratory-based results, field studies indicate that N_2_-fixation rates by unicellular diazotrophs increase with decreasing depth and increasing light in upwelling water where NO_3_^−^ concentrations are high [19], [20]. *Trichodesmium* blooms are also frequently observed in upwelling regions that are known to have high NO_3_^−^ concentrations [21]. Lastly, Deutsch et al. [22] presented a model proposing that N_2_-fixation rates might be very high in the Peru upwelling system, based on the distribution of phosphorus, despite high concentrations of NO_3_^−^ in this region. The general picture of how fixed N sources such as NO_3_^−^ control N_2_ fixation is still unclear.

In the context of these recent laboratory, field and modeling studies, we asked how the growth rate, as controlled by light, influences preferences for nitrogen substrates (e.g. NH_4_^+^, NO_3_^−^ and N_2_) to support growth of the unicellular N_2_ fixer *Crocosphaera watsonii*. Our data indicate that the N-source utilization ratio (NO_3_^−^:N_2_) changes in a predictable manner as a function of cell growth. We present experiments suggesting that three key parameters are necessary to determine how fixed N controls N_2_-fixation rates by *Crocosphaera watsonii*: 1) the cellular demand for N, which is largely controlled by the growth rate, 2) the light-specific cellular-assimilation kinetics of the various forms of N (e.g. V_max_) and 3) the relative concentrations of the various forms of N. Our basic model relies on the tenet that light energy is the driver of photoautotrophic growth rates while substrates such as NO_3_^−^, N_2_, PO_4_^3−^ etc. do not drive growth but serve as nutrient supports. Thus, a gradient in the light-energy supply rate creates a gradient in the demand for nitrogen to support growth and a gradient in the ratio of nutrient assimilation rates of various nutrient substrates. Our conceptual model may serve as a framework to understand how fixed N availability controls N_2_ fixation by oceanic diazotrophs. In light of expected future increases in anthropogenic fixed N inputs to both the coastal and open ocean [23], [24], these studies are needed to improve both physiological models and biogeochemical estimates of global biological N_2_ fixation and overall predictions of primary production trends over the next century [10], [25], [26].

## Materials and Methods

We investigated short-term and long-term effects of fixed N on N_2_-fixation rates by *C. watsonii* cultures (strain WH0003) in which growth rates were controlled by different light levels. In preparation for both short- and long-term experiments, *C. watsonii* was pre-acclimated to light environments by growing cultures in triplicate 1-L polycarbonate bottles at 25 and 175 µmol quanta m^−2^ s^−1^ and 28°C, on a 12∶12 hour light:dark cycle for 5 or more generations (as in other laboratory culture experiments; Berman-Frank et al. [18]) with an artificial seawater medium prepared according to the YBCII recipe of Chen et al. [27]. Trace metals (FeCl_3_·6H_2_O 4.50×10^−7^ M, MnCl_2_·4H_2_O 1.21×10^−7^ M, NaMoO_4_·2H_2_O 1.00×10^−7^ M, ZnSO4·7H_2_O 7.97×10^−8^ M, CoCl_2_·6H_2_O 5.03×10^−8^ M) and vitamins (Thiamine 2.96×10^−7^ M, B_12_ 3.96×10^−10^ M, Biotin 2.50×10^−9^ M) were added with the dilution medium [28] with 4 µM phosphate added as HNa_2_PO_4_. Cultures were grown with a semi-continuous culturing method as in other studies [14], [18], [29], [30], [31], [32] by diluting cultures every 3 days. Cultures were diluted by enumerating cells and calculating a dilution factor to achieve a target culture cell density of 20×10^3^ cells mL^−1^. We determined culture cell densities by agitating cultures just prior to collecting 5 ml of culture and enumerating live cells from subsamples microscopically. Although we did not continuously stir cultures, we did not observe cells or biomass sticking to the sides of the bottles. We calculated growth rates (*µ*) in between 3-day dilution periods with N_T_ = N_0_e*^µ^* ^T^, where N_0_ is the cell density at the beginning of a 3-day period (T) and N_T_ is the cell density at the end of the period.

### Short-term exposures

Initially, we exposed *Crocosphaera* to range of NH_4_^+^ concentrations for a short amount of time to gather basic information about how fixed N inhibits N_2_ fixation as a function of light-limited growth. We selected NH_4_^+^ because it has a high maximum uptake rate (V_max_) relative to other sources of fixed N in *Trichodesmium* [29]. Once we had collected data using NH_4_^+^ as an inhibitor, we repeated the short-term experimental design using NO_3_^−^ as the inhibitor. In short-term exposures, 50 mL samples were collected in 80 mL vials from each replicate culture and exposed to a range of NH_4_^+^ concentrations (0.2–2.0 µM, added as NH_4_Cl) and NO_3_^−^ (0.5–40 µM, added as NaNO_3_^−^; n = 3 for each treatment concentration of NH_4_^+^ or NO_3_^−^) just before the beginning of the dark period, approximately 3 hours before measurable ethylene concentrations accumulated. Replicates without added NH_4_^+^ or NO_3_^−^ served as controls. We estimated N_2_-fixation rates by injecting 4 mL acetylene into 30 mL headspace of the sample vials and measuring ethylene accumulation in 200 µl of the headspace over the 12-hour dark period with a gas chromatograph (model: GC-8A, Shimadzu Scientific Instruments, Columbia, MD, USA) [5], [6]. We used a 4∶1 ratio of N_2_:acetylene reduction to estimate N_2_-fixation rates [33]. Background ethylene concentrations in the acetylene source were small and subtracted from ethylene accumulation measurements. From each culture replicate, 100 mL were filtered onto combusted GF/F filters (500°C, 5 h), dried at 80°C, compressed into pellets and analyzed with an elemental analyzer (Costech instruments, model 4010) [5], [6]. The concentrations of particulate organic N were similar between cultures at the initiation of the short-term experiment (PN_lowlight_ = 4.3±0.6 µmoles N L^−1^; PN_highlight_ = 5.5±0.7 µmoles N L^−1^).

### Long-term exposures

Based on results from our initial short-term experiment with NO_3_^−^, we decided to expose *Crocosphaera* to NO_3_^−^ for a longer time period to determine if long-term exposures elicited a different response relative to that in the short-term exposure. In long-term exposures to NO_3_^−^, *C. watsonii* was pre-acclimated to experimental conditions in semi-continuous cultures using NO_3_^−^ as a fixed N source (added as 30 µM NaNO_3_), in parallel with control cultures growing without an added fixed N source. Particulate organic N of cultures was maintained at similar concentrations by semi-continuous dilution between the control (PN_lowlight_ = 6.6±3.3 µmoles N L^−1^; PN_highlight_ = 7.0±0.8 µmoles N L^−1^) and added NO_3_^−^ treatments (PN_lowlight_ = 6.7±0.9 µmoles N L^−1^; PN_highlight_ = 7.9±0.5 µmoles N L^−1^). We measured N_2_-fixation rates in 50 mL samples from each culture replicate with the acetylene reduction assay as described above at three experimental time points (Table 1). For estimates of NO_3_^−^ concentrations, we passed 20 mL of culture through a 0.45 µm syringe filter and NO_3_^−^ was measured by the analytical laboratory at the Marine Science Institute, University of California, Santa Barbara, CA, USA. We collected samples to measure the concentration of NO_3_^−^ from culture replicates 18 h after the last dilution of cultures (initial measurement) and either 48 h (high-light treatment) or 96 h (low-light treatment) after the initial measurement. To estimate cellular NO_3_^−^-assimilation rates, we normalized diminishing NO_3_^−^ concentrations during this time to culture cell concentrations that were calculated at the mid-point between these two time points using the growth rate. We did not examine a long-term response to NH_4_^+^ exposure primarily because it generally represents a small portion of fixed N relative to concentrations of NO_3_^−^ in many natural oceanic waters.

**Table 1:** Measurements of culture cell density (cells L^−1^×10^6^), dissolved nitrate+nitrite concentrations (NO_3_^−^ + NO_2_^−^, µmol L^−1^) and N_2_-fixation rates (fmol cell^−1^ hr^−1^) at different time points (hours since culture dilution) in cultures used in the short- and long-term exposure experiments.

Light intensity				Cells	[NO_3_^−^+NO_2_^−^]	N_2_
25 µmol quanta m^−2^ s^−1^						
	Short-term			38.4±4.8	0.15±0.05	
	Long-term					
		N_2_ only				
			T_18h_	25.8±0.9	0.13±0.07	13.6±8.1
			T_66h_	37.9±3.1	n.d.	65.2±4.2
			T_114h_	64.8±2.1	0.06±0.05	55.1±1.8
		+NO_3_^−^ (30 µM)				
			T_18h_	23.4±2.2	27.6±0.00	10.9±6.3
			T_66h_	36.4±2.9	25.8*±0.3	25.5±5.1
			T_114h_	59.7±5.9	23±0.7	23.8±1.8
175 µmol quanta m^−2^ s^−1^						
	Short-term			31±3.3	0.16±0.05	
	Long-term					
		N_2_ only				
			T_18h_	29.9±1.6	0.29±0.03	114.6±1.9
			T_42h_	60.4±1.3	n.d.	135.4±1.2
			T_66h_	117±12.1	0.05±0.01	115.4±12.5
		+NO_3_^−^ (30 µM)				
			T_18h_	33.8±1.3	28.7±0.5	105.5±1.5
			T_42h_	76.5±6.5	24.8*±0.3	127.7±5.6
			T_66h_	191.4±13.6	16.7±1.4	103.1±1.0

*calculated NO_3_^−^ concentrations.

Error (±) represents the standard deviation on 3 culture replicates.

## Results

We observed large differences in growth rates of *C. watsonii* between light treatments. In control cultures growing on N_2_ only, growth was significantly lower in low-light acclimated cultures (25 µmol quanta m^−2^ s^−1^; 0.23±0.02 d^−1^) relative to cultures growing under higher light (175 µmol quanta m^−2^ s^−1^, 0.68±0.03 d^−1^; t-test, *p*<0.05). The controlling effects of NH_4_^+^ and NO_3_^−^ on N_2_ fixation were different in short-term exposures, but varied as a function of growth rate. In addition, the effect of NO_3_^−^ on N_2_ fixation was similar between short and long-term exposures.

### Short-term exposures

**Figure 1:**
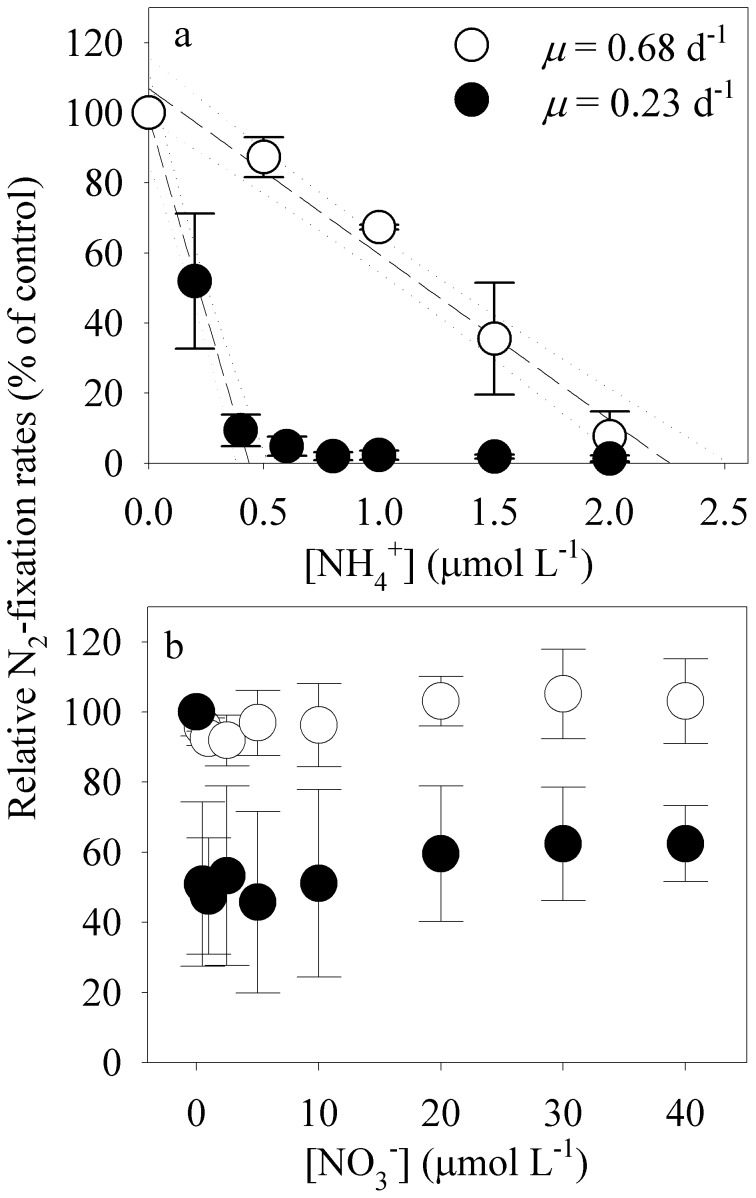
Short-term inhibitory effects of ammonium (NH_4_^+^, 0–1.5 µmol L^−1^) and nitrate (NO_3_^−^, 0–40 µmol L^−1^) on N_2_ fixation by *Crocosphaera watsonii* (WH0003) (percent of control with no added nitrogen). Cultures were grown in steady state under high light (175 µmol quanta m^−2^ s^−1^, growth rate (*µ*) = 0.68 d^−1^, open symbols) and low light (25 µmol quanta m^−2^ s^−1^, *µ* = 0.23 d^−1^, closed symbols) before adding nitrogen. Error bars represent standard deviations on means from 3 culture replicates.

In slow-growing cultures acclimated to low light, short-term additions of 0.4 µM NH_4_^+^ inhibited N_2_-fixation rates to <10% of rates in control treatments without added NH_4_^+^ (Fig. 1a). In faster-growing cultures acclimated to 175 µmol quanta m^−2^ s^−1^, with biomass concentrations equivalent to those in low-light cultures (Table 1), short-term exposure to five times as much NH_4_^+^ (2.0 µM) was needed to achieve the same inhibitory effect on N_2_ fixation (Fig. 1a). The short-term inhibitory effects of NO_3_^−^ on N_2_ fixation also varied as a function of growth rate. In slow-growing, low-light acclimated cultures, short-term exposure to NO_3_^−^ reduced mean N_2_-fixation rates by ∼47–62% relative to rates in control treatments without added NO_3_^−^ (Fig. 1b). In fast-growing cultures acclimated to high light, however, short-term additions of NO_3_^−^ at any concentration up to 40 µM did not inhibit mean N_2_-fixation rates by more than 9%, relative to N_2_-fixation rates in control cultures without added NO_3_^−^ (Fig. 1b).

### Long-term exposures

**Figure 2:**
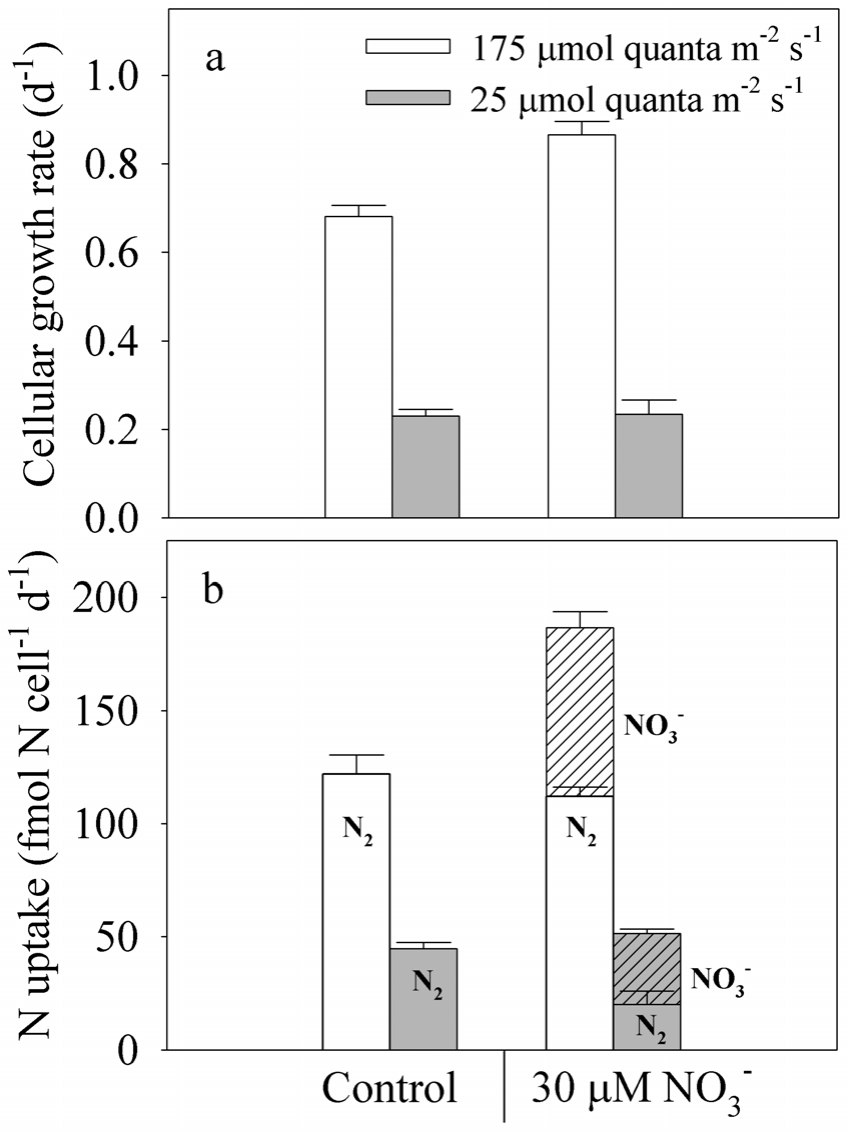
Effects of long-term exposure to 30 µmol L^−1^ nitrate (NO_3_^−^). (a) cellular growth rates and (b) nitrogen-assimilation rates of *Crocosphaera watsonii* (WH0003) acclimated to high light (175 µmol quanta m^−2^ s^−1^) and low light (25 µmol quanta m^−2^ s^−1^). (b) N_2_-fixation rates (solid bars) are overlain on total N assimilation (N_2_ + NO_3_^−^ assimilation, hashed bars). Control cultures did not receive added NO_3_^−^. Error bars represent standard deviations on means from 3 culture replicates.

**Figure 3:**
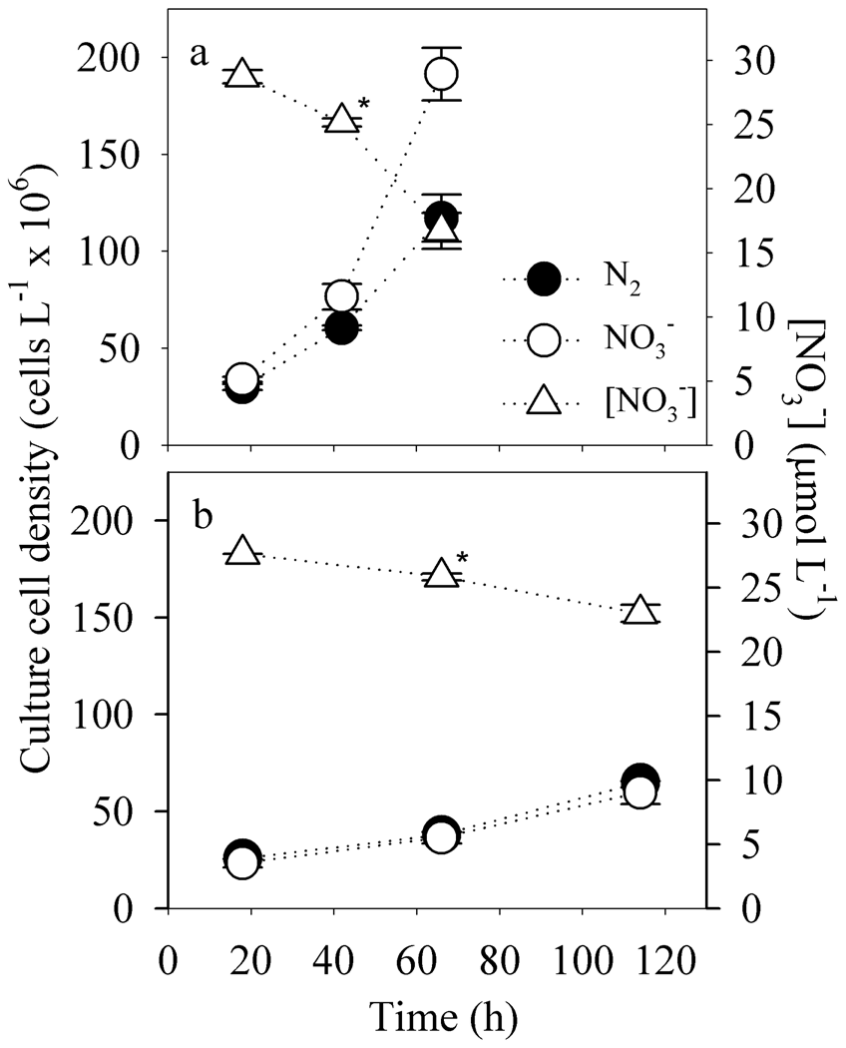
Changes in nitrate (triangles) and cell concentrations (circles) in cultures of *Crocosphaera watsonii* (WH0003) in long-term exposure experiments. Cultures were grown in steady state under (a) high light (175 µmol quanta m^−2^ s^−1^) and (b) low light (25 µmol quanta m^−2^ s^−1^) with added nitrate (30 µmol L^−1^; open symbols) or with N_2_ only (closed symbols). *Calculated NO_3_^−^ concentrations (see Methods section for details). Error bars represent standard deviations on means from 3 culture replicates.

In high-light-acclimated cultures, long-term exposure to 30 µM NO_3_^−^ yielded significantly higher growth rates (*µ* = 0.87 d^−1^) than those in control cultures without added NO_3_^−^ (*µ* = 0.68 d^−1^; *p*<0.05), indicating that growth was limited by the N_2_-assimilation rate (Fig. 2a). Diminishing NO_3_^−^ concentrations over time suggested that NO_3_^−^-assimilation rates in fast-growing cultures (*µ* = 0.87 d^−1^) were 2.8 times higher than those in slow-growing cultures (*µ* = 0.23 d^−1^; Fig. 3a; *p*<0.05), but the contribution of NO_3_^−^ to the total daily N assimilation still varied as a function of growth rate. In high-light-acclimated cultures exposed to NO_3_^−^ (*µ* = 0.87 d^−1^), NO_3_^−^ assimilation represented 40% of the total daily N assimilation while N_2_ assimilation represented 60% (Fig. 2b). When combined, NO_3_^−^ and N_2_ assimilation yielded a higher total daily N-assimilation rate (187 fmol N cell^−1^ d^−1^) than that in the control treatment growing on N_2_ only (122 fmol N cell^−1^ d^−1^; *p*<0.05; Fig. 2b). Furthermore, N_2_-fixation rates in cultures with added NO_3_^−^ were not significantly different than those in control cultures without NO_3_^−^ (*p*<0.05; Fig. 2b).

**Figure 4:**
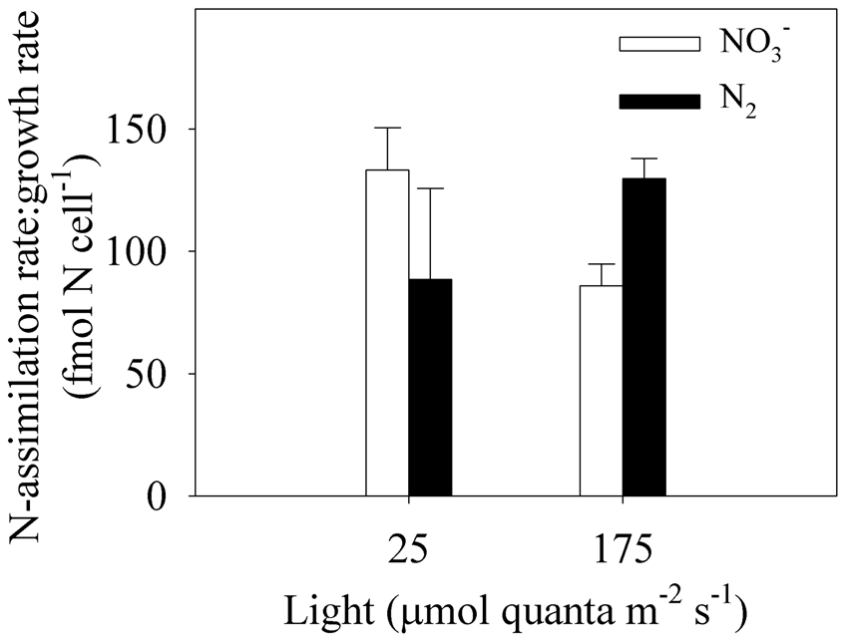
Growth-specific assimilation rates of nitrate (NO_3_^−^; open bars) and dinitrogen (N_2_; closed bars) in cultures of *C. watsonii* (WH0003) with added NO_3_^−^ (30 µmol L^−1^). Growth-specific NO_3_^−^ and N_2_-assimilation rates change inversely relative to each other as a function of light-limited growth. Error bars represent standard deviations on means from 3 culture replicates.

Under low light, long-term exposure to 30 µM NO_3_^−^ did not support faster growth rates (Fig. 2a, 3b) even though NO_3_^−^-uptake supported 61% of the total daily N assimilation. Instead, N_2_-fixation rates were reduced by 55% relative to those in cultures without added NO_3_^−^ (*p*<0.05; Fig. 2a). Thus, in cultures that were grown with NO_3_^−^, there was a clear shift in the ratio of N source utilization where growth-specific NO_3_^−^-assimilation rates increased by 55% with decreasing light,while growth-specific N_2_-assimilation rates increased by 46% with increasing light (Fig. 4). In both the high- and low-light treatments with 30 µM NO_3_^−^ added, the concentration of NO_3_^−^ was high (>16 µmol NO_3_^−^ L^−1^) throughout the entire 66 h or 114 h sampling period (Fig. 3).

## Discussion

Our main finding is that N-source utilization by *C. watsonii* varied as a function of the growth rate, which we controlled in our experiments with the supply of light energy. Thus, we interpret the variation in N-source utilization (e.g. NO_3_^−^:N_2_ or NH_4_^+^:N_2_) to be caused by a gradient in the demand for nitrogen as a substrate to support cell division. This N-source utilization ratio seems to change as a function of energy supply and growth rate because of differences in uptake kinetics between N sources (e.g. V_maxNO3−_: V_maxN2_) and energy requirements for the reduction and assimilation of each N source.

In our short-term exposure experiment with NH_4_^+^, fast-growing cultures of *C. watsonii* (*µ* = 0.68 d^−1^) needed a much higher concentration of NH_4_^+^ (5x) to satisfy the nitrogen substrate demand relative to slow-growing cultures (*µ* = 0.23 d^−1^; Fig. 1a). An alternate way to view this relationship is that as the amount of the added NH_4_^+^ decreased, increasing amounts of N_2_ were fixed to satisfy the remaining nitrogen demand to support cell growth (Fig. 1). These results suggest that the magnitude of assimilation of various N substrates depends on the cellular N demand that is needed to support the light-controlled growth rate relative to the light-specific cellular assimilation rate kinetics of each N source. Thus, we propose that when the light-controlled, growth-modulated demand for N exceeds the cellular-assimilation rate of NH_4_^+^ or NO_3_^−^, N_2_ fixation provides fixed N to fill the resulting N deficit.

The variable controlling effects of NO_3_^−^ and NH_4_^+^ on N_2_ fixation suggest that there are large differences in the assimilation kinetics of these different N species (Fig. 1). Under low light, low concentrations of NO_3_^−^ and NH_4_^+^ (0.5 µM) had maximum inhibitory effects on N_2_ fixation, suggesting that the half-saturation constants (K_s_) with respect to NO_3_^−^ and NH_4_^+^ are similar for *C. watsonii*. The incomplete inhibitory effect of NO_3_^−^ on N_2_-fixation rates even at high concentrations of NO_3_^−^ (Fig. 1b, 3b), however, suggests that the maximum NO_3_^−^-assimilation rate (V_max_) by *C. watsonii* (WH0003) is low relative to that of NH_4_^+^.

In our long-term experiment, we pre-acclimated *Crocosphaera* with high NO_3_^−^ concentrations (∼15–30 µM, Fig. 3) for 5 or more generations before sampling cultures over a 48–96 h period. In these long-term exposures to NO_3_^−^, we measured residual NO_3_^−^-concentrations in the culture medium to estimate the cellular NO_3_^−^-assimilation rate. The ratio of NO_3_^−^ -assimilation:N_2_ fixation varied as a function of energy supply and growth (Fig. 2), further supporting these variables as controls of fixed N inhibition of N_2_ fixation. Exposure to NO_3_^−^ did not affect N_2_ fixation by fast-growing cultures of *C. watsonii*, yet NO_3_^−^ comprised 40% of the total daily N, thereby supporting growth rates that were 27% higher than those in control cultures without added NO_3_^−^ (Fig. 2b). Thus, the growth of high-light cultures of *C. watsonii*, similar to *Cyanothece*, another marine unicellular N_2_ fixer [34], was clearly limited by the N_2_-assimilation rate, as the addition of 30 µM NO_3_^−^ supported higher growth rates (Fig. 2a).

These results indicate that growth rates of *C. watsonii* benefits from assimilating multiple N sources simultaneously, as individual assimilation rates of N_2_ or NO_3_^−^ alone cannot support maximum growth rates in high-light environments. Under low light, NO_3_^−^-assimilation did not support faster growth as it did under high light, but instead comprised 61% of the total daily assimilated N (Fig. 2). This higher contribution of NO_3_^−^ to the total N demand inhibited N_2_ fixation by 55% relative to rates in control cultures without added NO_3_^−^. Thus, we conclude that the inhibitory effect of NO_3_^−^ on N_2_ fixation by *C. watsonii* varies as a function of energy supply and growth rate.

Although we did not separate the direct effect of light-energy supply and growth rate in our long-term experiment, our analyses of the short-term effects of NH_4_^+^ and NO_3_^−^ exposure on N_2_ fixation were done only during dark hours when *Crocosphaera* fixes N_2_. Thus, *Crocosphaera* offers a unique advantage in comparison with *Trichodesmium* (which fixes CO_2_ and N_2_ simultaneously in the light) because it is possible to separate direct effects of light-energy supply from the effects of the light-limited growth rate on N-source utilization preferences. Future experiments might consider experiments that separate these effects by modulating growth rates in other ways.

The assimilation rates of the various chemical forms of N (e.g. NH_4_^+^, NO_3_^−^, N_2_) seem to be dictated in part by the energetic cost of reduction [16]. Many phytoplankton species are known to assimilate NH_4_^+^ more easily than NO_3_^−^ because of the lower energetic investment associated with assimilating NH_4_^+^ [35]. Although N-uptake kinetics have not been described for *C. watsonii*, Mulholland et al. [29] documented a maximum uptake rate for NH_4_^+^ by *Trichodesmium* that was presumably more than an order of magnitude higher than that for NO_3_^−^. Based on the relatively weak inhibitory effect of NO_3_^−^ on N_2_ fixation by *C. watsonii* relative to that observed for NH_4_^+^ (Fig. 1, 3), we infer that the maximum assimilation rate of NO_3_^−^ by *C. watsonii* (V_max_) must be considerably lower than that of NH_4_^+^.

Although NH_4_^+^ assimilation carries a cost associated with transport across the cell membrane, it is generally thought to be less expensive to assimilate than NO_3_^−^ and N_2_ [36], [37] because of the high costs associated NO_3_^−^ and N_2_ assimilation, which must first be reduced to NH_4_^+^ before being assimilated onto glutamic acid (ΔG  = +69 Kcal mol N^−1^ for NO_3_^−^ and +87 Kcal mol N^−1^ for N_2_) [16]. A lower assimilation cost for NH_4_^+^ might afford a high V_max_ relative to that for more energetically expensive forms of nitrogen. Thus, the lower cost associated with NO_3_^−^ reduction to NH_4_^+^ relative to N_2_ reduction to NH_4_^+^ appears to benefit *C. watsonii* in a light-limited environment where growth is slow relative to a maximum NO_3_^−^-assimilation rate (Fig. 4). In a high-light environment, the maximum assimilation rate of NO_3_^−^ relative to the growth rate is reduced in comparison with that in low-light cultures (Fig. 4), where N_2_ supports a higher portion of the daily N demand for growth. Future studies should quantify NO_3_^−^-assimilation kinetics for N_2_ fixers and identify how they might change as a function of other environmental conditions.

In addition to the energetic costs for reducing NO_3_^−^ and N_2_, the difference between energetic and material investments associated with the production of assimilatory proteins such as nitrogenase and nitrate reductase may be at least partially responsible for the differential ratios of NO_3_^−^:N_2_ reduction as function of growth. Tradeoffs in energetic investments for NO_3_^−^ and N_2_ reduction may come from balancing differential cellular nitrogen demands that are associated with variable growth rates [38] or from the supply of light. Further separating the effect of light-energy supply from the effect of growth on the ratio of fixed N:N_2_ utilization may lead to a better understanding of the release of fixed N by diazotrophs [5], [33], [39], [40], [41].

Contrary to findings by Ohki et al. [12] that suggest a strong time dependence of exposure to NO_3_^−^, NH_4_^+^ and urea in controlling inhibitory effects on N_2_ fixation in *Trichodesmium*, we documented consistent inhibitory effects of NO_3_^−^ on N_2_ fixation of *Crocosphaera* regardless of the duration of exposure. The results presented by Ohki et al. [12] are difficult to interpret in a context of supply and demand for N, however, because growth rates between treatments were not defined.

Although previous studies have not discussed inhibitory effects of fixed N on N_2_ fixation in a context of the supply rate of fixed N relative to the growth-modulated demand for N, four relatively recent studies have collectively examined inhibitory effects of fixed N on N_2_ fixation in batch cultures of *Crocosphaera* and/or *Trichodesmium* growing under 30–40, 80, 128 and 180 µmol quanta m^−2^ s^−1^, all at 26 or 27°C [14], [15], [17], [42]. In batch cultures, the biomass concentration of the culture is important to consider because of the accelerating effect of increasing biomass on the rate of disappearance of NO_3_^−^ or NH_4_^+^. Interpretation of these studies in a context of the supply rate of fixed N relative to the growth-modulated demand for N is also difficult, mainly because biomass and/or growth rates between treatments were not defined during batch-mode growth.

In our experiments, we maintained constant exponential growth rates with a semi-continuous culturing method and we maintained equivalent biomass concentrations between treatments so that differences in NH_4_^+^ and NO_3_^−^ drawdown due to biomass differences would not affect cellular N_2_-fixation rates between treatments and between time points (Fig. 3; Table 1). In addition to our experiments with *Crocosphaera*, all of these previous studies indicate that NO_3_^−^ and/or NH_4_^+^ have controlling effects on N_2_ fixation by oceanic N_2_ fixers. Future studies that examine N-source preferences should focus on growth-modulated controls of fixed N on N_2_ fixation in both *Trichodesmium* and *Crocosphaera*. Although we presume that this model would be similar for *Trichodesmium*, there may be unforeseeable differences due to the major differences between the physiological mechanisms that these species use to separate oxygen generated by photosynthesis from the nitrogenase enzyme; *Trichodesmium* seems to use a spatial separation mechanism, as it fixes both inorganic carbon and N_2_ during the light period. In contrast, *Crocosphaera* uses a temporal separation mechanism, as it stores fixed carbon during the light period and respires it for energy during the night to fuel N_2_ fixation in the dark, similar to the unicellular strategy described by Berman-Frank et al. [18].

In the open ocean, the primary limiting nutrients for growth of N_2_-fixing cyanobacteria are iron (Fe) and phosphorus (P) [43], [44]. In combination with light, Fe and P have an indirect effect on N demand through their support of cellular growth. Capone and Knapp [8] originally proposed that the N:P ratio is important in controlling N_2_-fixation rates, and recently Ward et al. [11] suggested that the N:Fe ratio is a dominant controlling factor of marine N_2_ fixation. Our basic model suggests that the ratio of N:X is important in controlling N_2_-fixation rates where “X” is a resource that influences growth rates (such as light, P and Fe), and thereby, the demand for N. Laboratory data support this, where high concentrations of P supported high N_2_-fixation rates relative to cultures with lower P concentrations, despite equivalent N:P supply ratios [15]. In a modeling study, Ward et al. [11] demonstrated that the N:P supply ratio is a secondary factor in defining boundaries of N_2_ fixation, while the N:Fe supply ratio is more important in an ecological context through competitive interactions with non-N_2_-fixing phytoplankton. Further, Garcia et al. [32] suggest that the Fe:P supply ratio may be more important in controlling N_2_ fixation than the absolute concentration of either of these limiting nutrients. Collectively, these studies suggest that links between C, N, P and Fe biogeochemical cycles depend on the relative supply of each of these nutrients and our study further suggests that the energy-supply rate or the growth rate modulates interactions between these nutrients.

Our study indicates that global models of marine biological N_2_ fixation should consider an interaction between assimilation kinetics of fixed N and a growth-modulated demand for N. Although our study did not focus on how *Crocosphaera* might respond in the natural environment, our data provide a framework around which future studies might structure investigations of N-source preferences by natural communities of N_2_ fixers. Reactive nitrogen from atmospheric sources and agricultural runoff are expected to increase in the future and the effects of increased N input to the oceans on phytoplankton communities is uncertain [23], [24], [45]. Thus, a clear understanding of how reactive nitrogen affects N_2_ fixation is needed to support predictions of how phytoplankton communities will change.

Two other relevant environmental factors that will certainly influence growth of N_2_ fixers in the future are CO_2_ and temperature [4], [5], [6], [7], [30], [34]. Both of these factors are predicted to increase, and will likely influence the controlling effects of fixed N on N_2_ fixation through their effects on growth rates. Thus, our basic framework potentially has far-reaching implications for both current estimates of oceanic N_2_ fixation, and for estimates of N_2_-fixation rates that are likely to exist in the future surface oceans [3].
